# Enhanced Nutcracker Optimization Algorithm with Hyperbolic Sine–Cosine Improvement for UAV Path Planning

**DOI:** 10.3390/biomimetics9120757

**Published:** 2024-12-12

**Authors:** Shuhao Jiang, Shengliang Cui, Haoran Song, Yizi Lu, Yong Zhang

**Affiliations:** School of Information Engineering, Tianjin University of Commerce, Tianjin 300134, China; cuiliang@stumail.tjcu.edu.cn (S.C.); songhaoran@stu.tjcu.edu.cn (H.S.); 15036671877@163.com (Y.L.); zhangyong@163.com (Y.Z.)

**Keywords:** nutcracker optimizer, path planning, metaheuristic, unmanned aerial vehicle, a sinh cosh optimizer

## Abstract

Three-dimensional (3D) path planning is a crucial technology for ensuring the efficient and safe flight of UAVs in complex environments. Traditional path planning algorithms often find it challenging to navigate complex obstacle environments, making it challenging to quickly identify the optimal path. To address these challenges, this paper introduces a Nutcracker Optimizer integrated with Hyperbolic Sine–Cosine (ISCHNOA). First, the exploitation process of the sinh cosh optimizer is incorporated into the foraging strategy to enhance the efficiency of nutcracker in locating high-quality food sources within the search area. Secondly, a nonlinear function is designed to improve the algorithm’s convergence speed. Finally, a sinh cosh optimizer that incorporates historical positions and dynamic factors is introduced to enhance the influence of the optimal position on the search process, thereby improving the accuracy of the nutcracker in retrieving stored food. In this paper, the performance of the ISCHNOA algorithm is tested using 14 classical benchmark test functions as well as the CEC2014 and CEC2020 suites and applied to UAV path planning models. The experimental results demonstrate that the ISCHNOA algorithm outperforms the other algorithms across the three test suites, with the total cost of the planned UAV paths being lower.

## 1. Introduction

Drone technology is rapidly maturing, exhibiting high flexibility, efficiency, and autonomy. Its range of applications is expanding, allowing drones to effectively replace humans in executing high-risk tasks. Among them, searching for trapped people in disaster areas is one of the important application areas of drones. Search and rescue missions can be extremely challenging for rescue teams due to terrain constraints. However, drones are better suited for these tasks because they can navigate around ground obstacles and conduct searches significantly faster than humans. Effective and efficient path planning not only reduces the cost of search and rescue operations but also significantly enhances their overall efficiency. The UAV path planning problem can be defined as an optimization challenge, with the primary objective of finding the most cost-effective and efficient route between the starting and ending points while meeting all constraints [1]. In contrast to manual control of the UAV flight path by the operator [2], employing metaheuristic algorithms for UAV flight path planning can yield significantly higher efficiency and accuracy [3]. However, in areas with dense obstacles, drones often face challenges in finding the optimal path and may even risk collisions. Therefore, in recent years, many researchers have extensively explored the UAV path planning problem, aiming to identify flight routes that minimize costs, enhance safety, and reduce travel time [4].

UAV search and rescue is fundamentally a three-dimensional path planning problem, and the main algorithms for optimizing UAV paths include traditional optimization methods and metaheuristic algorithms. Traditional optimization algorithms are techniques utilized to determine the optimal solution for a given problem, including Dijkstra’s algorithm [5], integer programming [6], and linear programming [7]. Dijkstra’s algorithm can accurately find the shortest path; however, it requires traversing all nodes in the graph and cannot handle negative weights. Consequently, it often requires enhancement through supplementary algorithms or improvements to boost its performance. Since traditional Dijkstra’s algorithm struggles to handle problems with complex constraints, Subaselvi Sundarraj et al. [8] proposed an improved particle swarm optimization (PSO) algorithm that integrates weight control with Dijkstra’s algorithm. Deng et al. [9] enhanced Dijkstra’s algorithm by applying the integral theorem to improve its performance in handling uncertain edge weights. However, this improvement yields favorable results only in specific scenarios, and the algorithm’s computational efficiency remains inadequate in more complex environments. Linear programming methods, such as the simplex and interior point algorithms, can guarantee the existence and uniqueness of a solution. However, these methods often struggle with uncertain problems, and the optimization results may not always be globally optimal when dealing with complex or highly nonlinear issues. Therefore, Jiang et al. [10] combined linear programming, fuzzy clustering, and pigeon optimization algorithms to enhance the performance of linear programming in addressing nonlinear problems. The integrated approach significantly improves both optimization effectiveness and computational efficiency. However, it still remains susceptible to falling into local optima. Cheng et al. [11] proposed a mixed logical linear programming algorithm to enhance both the computational efficiency and the solution quality of linear programming. In addition, Kvitko et al. [12] integrated chaos theory with neural network modeling, offering a novel approach to the path planning problem. Moysis et al. [13] introduced an innovative 3D path planning method that leverages chaotic mapping to generate pseudo-random bit sequences, enhancing both the randomness and efficiency of path design. Although traditional algorithms have made significant progress, they typically require the objective function to be continuously differentiable and are prone to getting stuck in local optima [14]. These limitations have prompted researchers to prefer metaheuristic algorithms.

Metaheuristic optimization algorithms are inspired by natural and physical phenomena. Generally, these algorithms can be classified into four categories [15]: group intelligence-based algorithms, physics-based algorithms, human-based algorithms, and evolution-based algorithms. Metaheuristic algorithms are more stochastic than traditional optimization algorithms, which can adjust their parameters based on specific problems. This inherent stochasticity allows them to effectively tackle complex nonlinear issues [16] and has made them popular among researchers for their ability to yield satisfactory solutions. Examples include optimizing real-valued parameters and constrained engineering problems [17], energy management [18], flow shop scheduling [19], brain tumor classification [20], biomedical feature selection [21], combinatorial optimization [22], supply chain management [23], wireless sensor network localization [24], and clustering [25]. Group intelligence algorithms are derived from simulating the social behaviors of swarming organisms, with particle swarm optimization [26] (PSO) being the most popular among researchers; this algorithm simulates the foraging behavior of birds and is characterized by a simple structure and fewer parameters [27]. Jiang et al. [28] divided the flock into several sub-flocks, with each sub-flock performing PSO-based updates and iterations simultaneously. At the later stages of iteration, each sub-flock shares its optimal position, significantly enhancing both the exploration and exploitation capabilities of PSO. However, it is evident that this improvement strategy does not focus on optimizing the convergence speed of PSO. Sun et al. [29] divided the bird flocks into main flocks and sub-flocks, with the main flocks tasked with conducting a random optimization search in the search space to maintain diversity, while the sub-flocks focused on exploring regions near the local optimum. The synergy between these two populations significantly enhances the convergence speed of the algorithm; however, the algorithm remains prone to falling into local optima. Shao et al. [30] designed an adaptive linear variation of acceleration coefficients and maximum velocities to introduce random mutations for low-quality particles, thereby improving solution quality and reducing the risk of falling into local optima. However, the convergence accuracy of this algorithm remains lower compared to more recent swarm intelligence algorithms. Meanwhile, to enhance the convergence accuracy of the algorithm, Jiang et al. [31] incorporated an information feedback model into the Flamingo Search Algorithm (FSA). However, the stability of this modified algorithm remains relatively weak. Therefore, [32] combined PSO, which incorporates three inertia weight coefficients, with the Symbiotic Organism Search (SOS) algorithm to improve both the convergence accuracy and stability of the algorithm. Evolution-based optimization algorithms identify near-optimal solutions by simulating the natural concept of survival of the fittest. Common approaches include the genetic algorithm (GA) [33] and differential evolution (DE) [34]. The genetic algorithm transforms the optimization problem into a process of chromosome crossover and recombination in biological evolution. However, it suffers from poor population diversity and is less suited for solving continuous problems. Krishna et al. [35] combined a genetic algorithm with the K-means algorithm and demonstrated the convergence of this hybrid approach. However, achieving accurate convergence remains a significant challenge. Physics-based optimization algorithms simulate physical phenomena, such as black-hole optimization (BH). Pashaei et al. [36] demonstrate that the binary black hole algorithm offers a notable improvement in computational complexity; however, it still depends on the choice of initial parameters. Human HBBO algorithms simulate the two phases of human exploration and exploitation. Liu et al. [37] proposed a particle swarm algorithm that incorporates human behavior, significantly enhancing both the convergence speed and accuracy of the algorithm. However, despite these improvements, the algorithm still struggles to guarantee the discovery of a globally optimal solution. From the above discussion, it is evident that most metaheuristic algorithms are prone to issues such as getting stuck in local optima, lacking sufficient population diversity, and exhibiting slow convergence.

Compared with other metaheuristic algorithms, the nutcracker optimizer [38] (NOA) is characterized by its simple structure, significant randomness, and a low tendency to get trapped in local optima. The NOA algorithm, developed by Abdel-Basset et al., is a population-based optimization method. Due to its outstanding optimization capabilities, it has captured the attention of researchers since its inception and has been utilized in multiple areas. Applications include solar PV model parameter extraction [39], the performance of the NOA on multi-objective problems [40], fresh produce distribution [41], detection of news truthfulness and reliability [42], power system scheduling [43], and medical image classification [44]. Despite its successful application across various fields, the nutcracker optimizer encounters challenges, including slow convergence rates and limited accuracy in convergence. SCHO [45] exhibits excellent exploitation capabilities while maintaining a strong balance between the exploration and exploitation processes. This study effectively integrates the SCHO algorithm into the nutcracker optimizer. This novel approach tackles the limitations of the NOA algorithm, such as inefficient searching and challenges in fully leveraging reference point information. The contributions of this paper are specified below:

1. To tackle the issue of randomized foraging strategies and wide search ranges that result in inefficient search, we integrate the SCHO algorithm to improve the search efficiency of the NOA algorithm by enhancing the foraging strategy to search for high-quality food.
2. To tackle the issue of rapid population diversity loss and slow convergence in storage strategies, we employ a nonlinear function to steer the search direction of the current optimal individual while simultaneously preserving population diversity in the later iterations to prevent convergence to a local optimum.
3. Recognizing that nutcrackers do not fully leverage the information regarding reference point locations, this paper implements an improved SCHO algorithm to incorporate this reference point information. This enhancement bolsters the collaborative search capabilities of the nutcracker populations.
4. The performance of the improved algorithm is assessed using 14 classical benchmark test functions, as well as the CEC2014 and CEC2020 test suites. Additionally, the optimized algorithm is applied to real map models with progressively increasing densities, thereby validating its effectiveness.

ISCHNOA demonstrates competitive performance not only against recently proposed NOA algorithms but also when compared to other algorithms that exhibit superior performance. The remainder of this paper is structured as follows: Section 2 provides a description of the UAV path planning problem. Section 3 presents the NOA algorithm. Section 4 introduces the ISCHNOA algorithm. Section 5 assesses the performance of the ISCHNOA algorithm and applies it to 3D maps in real-world scenarios. Section 6 provides a summary of the research.

## 2. Description of the Issue

The UAV path planning problem involves finding an optimal path from the start position to the goal position under a set of constraints. The optimization process, on the other hand, further refines this path to identify a near-optimal solution. Constraints can be represented by cost functions, which include path cost, threat cost, altitude cost, and smoothing cost [46]. These factors are considered collectively to ensure the smooth operation of UAVs in complex environments.

### 2.1. Path Cost

The path cost reflects the distance between the starting point and the destination. In this study, the UAV path is composed of multiple waypoints, with the coordinates of each waypoint denoted as $P_{ij}=\left(x_{ij},y_{ij},z_{ij}\right)$. The distance between two nodes is calculated using the Euclidean distance, denoted as $∥\vec{P_{i,j}P_{i,j+1}}∥$. The total path cost $F_{1}$ is mathematically modeled as shown in Equation (1).

(1) $$F_{1}=\sum_{j=1}^{n-1}∥\vec{P_{ij}P_{i,j+1}}∥$$

### 2.2. Threat Cost

**Figure 1:**
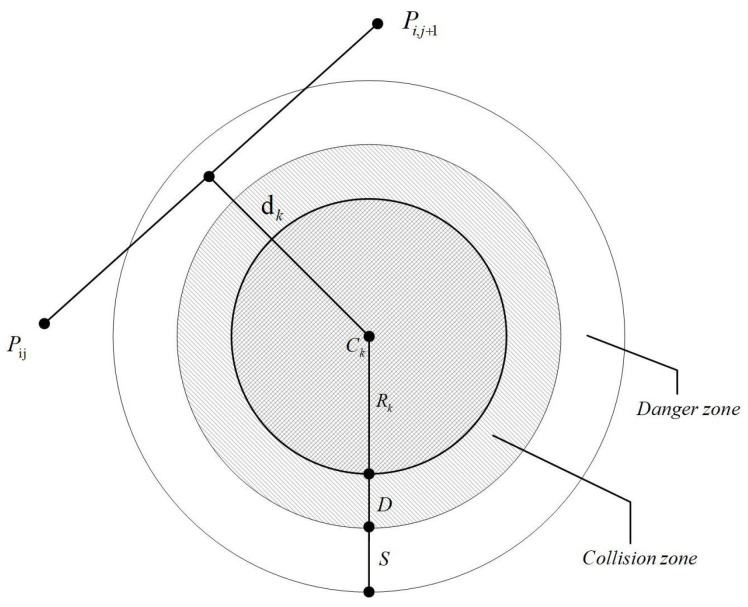
Threat cost visualization.

Threat costs refer to the potential risks of collision during drone flight. During flight, there is a risk of drones colliding with obstacles. Effective obstacle avoidance is a crucial factor in assessing the threat cost. The magnitude of the threat cost is closely related to the distance between the UAV and obstacles, allowing the entire flight area to be categorized into safe, threat, and collision zones. Figure 1 illustrates the connections between these three regions. When the flight path is within the safety region, its cost is zero. However, if the flight path enters the collision region, it becomes impossible to determine an optimal path. The threat cost $F_{2}$ is represented by Equation (2).

(2) $$\left\{\begin{array}{c} F_{2}\left(x_{i}\right)=\sum_{j=1}^{n-1}\sum_{k=1}^{K}T_{k}\left(\vec{P_{ij}P_{i,j+1}}\right)\\ T_{k}\left(\vec{P_{ij}P_{i,j+1}}\right)=\left\{\begin{array}{cc} \infty , & if\;{\begin{array}{c} d \end{array}}_{k}\leq D+R_{k}\\ \left(S+D+R_{k}\right)-d_{k} & if\;\begin{array}{c} D+R_{k}<d_{k}\leq S+D+R_{k} \end{array}\\ 0 & if\;\begin{array}{c} d_{k}\leq D+R_{k} \end{array} \end{array}\right. \end{array}\right.$$

The obstacles examined in this paper are regular cylinders. The number of obstacles is denoted by *K*, with the center of each obstacle denoted as $C_{k}$, the radius as $R_{k}$, and the diameter of the drone as *D*.

### 2.3. Altitude Cost

**Figure 2:**
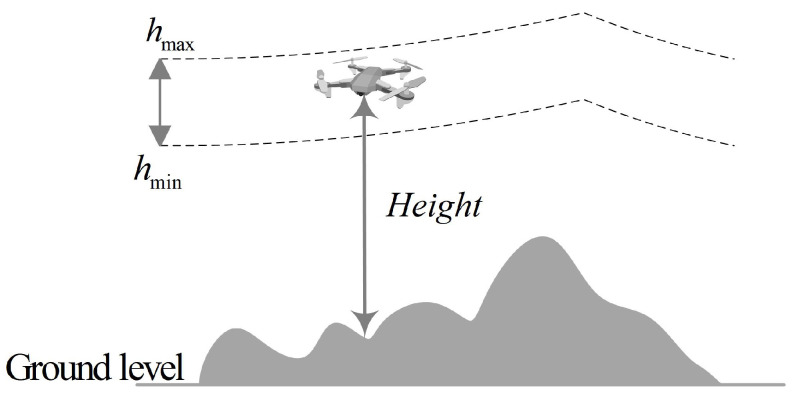
Flight altitude visualization.

Altitude costs refer to the additional expenses incurred during flight as a result of changes in altitude. Figure 2 illustrates that drone flight altitude must be maintained within a specified minimum and maximum range, beyond which penalties will be incurred. When drones fly at high altitudes, they become more susceptible to external environmental factors, which increases the risk of flight accidents. Conversely, flying at excessively low altitudes poses a danger of collisions with the ground. The altitude cost $F_{3}$ is represented by Equation (4).

(3) $$H_{\mathrm{ij}}=\left\{\begin{array}{cc} \left|h_{ij}-\frac{\left(h_{\max}+h_{\min}\right)}{2}\right|, & if\;\begin{array}{c} h_{\min}\leq h_{ij}\leq h_{\max} \end{array}\\ \infty , & otherwise \end{array}\right.$$

(4) $$F_{3}\left(x_{i}\right)=\sum_{j=1}^{n}H_{ij}$$

where the maximum and minimum flight altitudes are denoted as $h_{\max}$ and $h_{\min}$, respectively, and $h_{ij}$ indicates the UAV’s height above the ground.

### 2.4. Smoothing Cost

The smoothing cost is introduced in the path optimization process to minimize abrupt changes in curvature and steering angle of the flight path. Let three consecutive points be $P_{\mathrm{ij}}$, $P_{i,j+1}$, and $P_{i,j+2}$, with their projections onto the xoy plane denoted as $P_{\mathrm{ij}}^{\prime }$, $P_{i,j+1}^{\prime }$, and $P_{i,j+2}^{\prime }$, respectively. The vector between two consecutive points is calculated as shown in Equation (5).

(5) $$\vec{P_{ij}^{\prime }P_{i,j+1}^{\prime }}=\vec{k}\times \left(\vec{P_{ij}P_{i,j+1}}\times \vec{k}\right)$$

**Figure 3:**
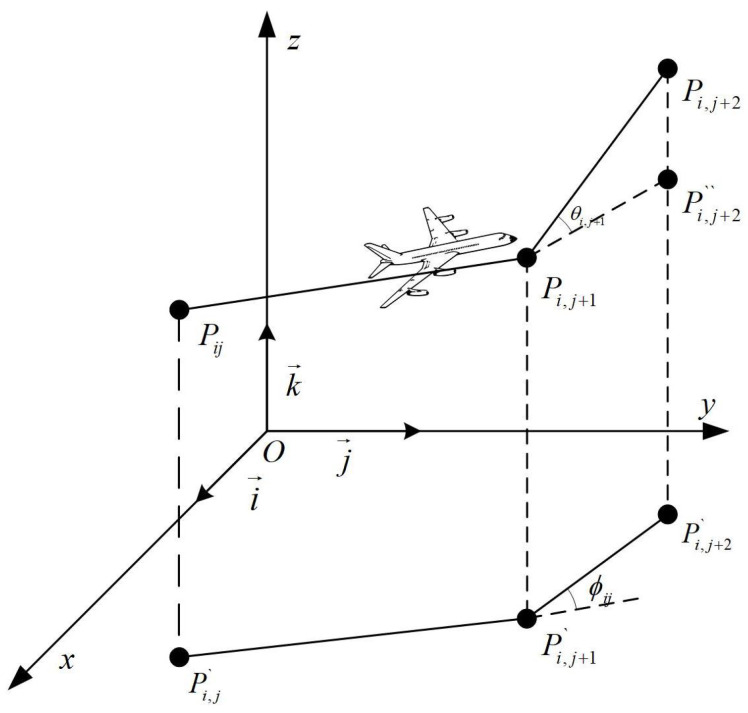
Turning and climbing angle calculation.

$\vec{k}$ is the unit vector in the Z-axis direction, while the steering and climb angles are designated as Φ and ψ, respectively. Figure 3 illustrates the steering angle and climb angle, defined in Equation (6) and Equation (7), respectively. The smoothing cost $F_{4}$ is mathematically modeled as shown in Equation (8).

(6) $$\Phi_{ij}=\arctan\left(\frac{∥\vec{P_{ij}^{\prime }P_{i,j+1}^{\prime }}\times \vec{P_{i,j+1}^{\prime }P_{i,j+2}^{\prime }}∥}{\vec{P_{ij}^{\prime }P_{i,j+1}^{\prime }}\cdot \vec{P_{i,j+1}^{\prime }P_{i,j+2}^{\prime }}}\right)$$

(7) $$\psi_{ij}=\arctan\left(\frac{z_{i,j+1}-z_{ij}}{∥\vec{P_{ij}^{\prime }P_{i,j+1}^{\prime }}∥}\right)$$

(8) $$F_{4}\left(x_{i}\right)=\omega_{1}\sum_{j=1}^{n-2}\Phi_{ij}+\omega_{2}\sum_{j=1}^{n-1}\left|\psi_{ij}-\psi_{i,j-1}\right|$$

where $\omega_{1}$ and $\omega_{2}$ are the penalty coefficients for the steering and climb angles, respectively.

### 2.5. Overall Cost Function

The total cost of ownership in UAV path planning is a crucial factor in ensuring mission success and safety. Incorporating these costs into the planning process can effectively reduce collision risk, optimize flight altitude, enhance energy efficiency, and minimize mission completion time. Considering these costs during the planning process can effectively reduce collision risk, optimize flight altitude, enhance energy efficiency, and shorten mission completion times, thereby better addressing the needs of various applications. The total cost function *F* is defined in Equation (9).

(9) $$F=\sum_{k=1}^{4}\alpha_{k}F_{k}\left(x_{i}\right)$$

where $\alpha_{1}$∼$\alpha_{4}$ represent the weighting coefficients for path cost, threat cost, height cost, and smoothing cost, respectively.

## 3. Overview of Nutcracker Optimizer

The NOA algorithm, a metaheuristic optimization algorithm, is inspired by the behaviors of nutcrackers throughout different seasons. In summer and fall, nutcrackers forage for high-quality food in open areas and store it away. In spring and winter, they retrieve their stored food by using information about reference points in their environment.

### 3.1. Population Initialization

Assuming that there are N individuals in the nutcracker population, the initialization formula is as in Equation (10).

(10) $${\vec{X}}_{i,j}^{t}=\left(u_{b}-u_{l}\right)*\vec{rand}+u_{l},\begin{array}{cc} & i=1,2,\cdots ,N,\; \end{array}j=1,2,\cdots ,D$$

where *t* represents the current moment, and $u_{b}$ and $u_{l}$ are the upper and lower bounds of the search region, respectively. *N* represents the number of nutcracker individuals in the population, *D* represents the dimension, and $\vec{rand}$ is a random vector in the interval [0, 1].

### 3.2. Foraging Strategy

In their foraging strategy, nutcrackers search for high-quality seeds within their foraging area. When they find high-quality food, they return it to their storage area using their own methods. However, if they encounter lower-quality seeds, the nutcracker will reselect their location and continue searching for better options. The overall foraging strategy is presented in Equation (11).

(11) $${\vec{X}}_{i}^{t+1}=\left\{\begin{array}{cc} X_{i,j}^{t} & if\;\begin{array}{c} \eta_{1}<\eta_{2} \end{array}\\ \left\{\begin{array}{cc} X_{m,j}^{t}+\gamma \cdot \left(X_{A,j}^{t}-X_{B,j}^{t}\right)+\mu \cdot \left(r_{1}^{2}\cdot u_{b,j}-u_{l,j}\right), & if\;\begin{array}{c} t\leq \frac{T}{2} \end{array}\\ X_{c,j}^{t}+\mu \cdot \left(X_{A,j}^{t}-X_{B,j}^{t}\right)+\mu \cdot \left(r_{2}<\delta \right)\left(r_{1}^{2}\cdot u_{b,j}-u_{l,j}\right), & Otherwise \end{array}\right. & otherwise \end{array}\right.$$

where ${\vec{X}}_{i}^{t+1}$ represents the position of the *i*-th nutcracker after updating in the current generation, ${\vec{X}}_{m,j}^{t}$ denotes the average position of the *j*-th dimensional nutcracker population in that generation, and ${\vec{X}}_{A,j}^{t}$ and ${\vec{X}}_{B,j}^{t}$ are two different individual nutcrackers randomly selected from the *j*-th dimensional population. Additionally, $u_{b,j}$ and $u_{l,j}$ define the upper and lower bounds of the *j*-th dimensional range of positions, $r_{1}$∼$r_{4}$, $\eta_{1}$ and $\eta_{6}$∼$\eta_{11}$ is a random number in the range of [0, 1], γ and $\eta_{3}$ is a random number generated by Levy flight, $\eta_{2}$ satisfies normal distribution, and μ is defined as shown in Equation (12).

(12) $$\mu =\left\{\begin{array}{cc} \eta_{1}, & r_{2}<r_{3}\\ \eta_{2}, & r_{3}<r_{4}\\ \eta_{3}, & r_{2}<r_{4} \end{array}\right.$$

### 3.3. Storage Strategy

In the food storage strategy, the nutcracker will transport high-quality seeds found during the exploration phase to the storage area. The overall storage strategy is presented in Equation (13).

(13) $${\vec{X}}_{i}^{t+1}=\left\{\begin{array}{cc} {\vec{X}}_{i}^{t}+\mu \cdot \left({\vec{X}}_{best}^{t}-{\vec{X}}_{i}^{t}\right)\cdot \left|\lambda \right|+r_{2}\cdot \left({\vec{X}}_{A}^{t}-{\vec{X}}_{B}^{t}\right) & if\;\begin{array}{c} \eta_{1}<\eta_{2} \end{array}\\ {\vec{X}}_{best}^{t}+\mu \cdot \left({\vec{X}}_{A}^{t}-{\vec{X}}_{B}^{t}\right) & if\;\begin{array}{c} \eta_{1}<\eta_{5} \end{array}\\ {\vec{X}}_{best}^{t}\cdot l & Otherwise \end{array}\right.$$

where ${\vec{X}}_{\mathrm{best}}^{t}$ represents the historically optimal position for the nutcracker, λ is a random number generated using Levy flight strategy, and *l* is a linearly decreasing function defined over the interval [0, 1], effectively aiding in avoiding the algorithm’s convergence to a local optimum. Meanwhile, Equation (14) helps maintain a balance between the foraging and storage strategies of the NOA algorithm.

(14) $$\vec{X_{i}^{t+1}}=\left\{\begin{array}{cc} Equation\;(11), & if\;\begin{array}{c} \phi >P_{a_{1}} \end{array}\\ Equation\;(13), & otherwise \end{array}\right.$$

where ϕ is a random number in the interval [0, 1] and $P_{a1}$ is a factor that decreases linearly from 1 to 0.

### 3.4. Caching Strategy

During the winter months, the nutcracker transitions from storage mode to cache mode. With its exceptional spatial memory, the nutcracker can remember the locations of reference points marked during the storage phase and efficiently locate stored seeds using these markers. There may be many reference point locations for individual marking of nutcrackers, but in this paper, only two reference points were selected for the study and are referred to as RP. RP can be represented by a matrix as in Equation (15).

(15) $$RPs=\left[\begin{array}{cc} \vec{RP_{1,1}^{t}} & \vec{RP_{1,2}^{t}}\\ \vdots & \vdots \\ \vec{RP_{i,1}^{t}} & \vec{RP_{i,2}^{t}}\\ \vdots & \vdots \\ \vec{RP_{N,1}^{t}} & \vec{RP_{N,2}^{t}}\\ \vdots & \vdots \end{array}\right]$$

where ${\vec{RP}}_{1,1}^{t}$ and ${\vec{RP}}_{1,2}^{t}$ are the first and second reference points of the nutcracker in generation *t*, respectively. The position initialization equations for the two reference points are shown in Equations (16) and (17).

(16) $${\vec{RP}}_{i,1}^{t}=\left\{\begin{array}{cc} {\vec{X}}_{i}^{t}+\alpha \cdot \cos\left(\theta \right)\cdot \left(\left({\vec{X}}_{A}^{t}-{\vec{X}}_{B}^{t}\right)\right)+\alpha \cdot RP, & if\;\begin{array}{c} \theta =\frac{\pi }{2} \end{array}\\ {\vec{X}}_{i}^{t}+\alpha \cdot \cos\left(\theta \right)\cdot \left(\left({\vec{X}}_{A}^{t}-{\vec{X}}_{B}^{t}\right)\right), & otherwise \end{array}\right.$$

(17) $${\vec{RP}}_{i,2}^{t}=\left\{\begin{array}{cc} {\vec{X}}_{i}^{t}+\left(\alpha \cdot \cos\left(\theta \right)\cdot \left(\left(\vec{u_{b}}-\vec{u_{l}}\right)\cdot \eta_{5}+\vec{u_{l}}\right)+\alpha \cdot RP\right)\cdot \vec{U}, & if\;\begin{array}{c} \theta =\frac{\pi }{2} \end{array}\\ {\vec{X}}_{i}^{t}+\alpha \cdot \cos\left(\theta \right)\cdot \left(\left(\vec{u_{b}}-\vec{u_{l}}\right)\cdot \eta_{5}+\vec{u_{l}}\right)\cdot \vec{U}, & otherwise \end{array}\right.$$

where ${\vec{RP}}_{i,1}^{t}$ represents row *i*, column 1 in the reference point matrix and ${\vec{RP}}_{i,2}^{t}$ represents row *i*, column 2 in the reference point matrix. $\vec{U}$ is a binary vector, and α is a function that decreases linearly from 1 to 0. The definitions are as in Equation (18) and Equation (19), respectively.

(18) $$\vec{U}=\left\{\begin{array}{cc} 1, & if\;\begin{array}{c} r_{3}<P_{rp} \end{array}\\ 0, & else \end{array}\right.$$

(19) $$\alpha =\left\{\begin{array}{cc} {\left(1-\frac{t}{T}\right)}^{2\cdot \frac{t}{T}}, & if\;\begin{array}{c} r_{2}>r_{3} \end{array}\\ {\left(\frac{t}{T}\right)}^{\frac{2}{t}} & else \end{array}\right.$$

where *t* and *T* represent the current iteration number and the maximum iteration number, respectively, and α is proposed to ensure the convergence speed of the NOA algorithm. As the iteration proceeds, the nutcracker explores regions that may be near the stored food and searches using reference point locations that suit its needs. The nutcracker’s new position is updated based on the relationship between the reference point and the fitness value of its current location, which is mathematically modeled as shown in Equation (20).

(20) $${\vec{X}}_{i}^{t+1,1}=\left\{\begin{array}{cc} \vec{X_{i}^{t}}, & if\;\begin{array}{c} f\left(\vec{X_{i}^{t}}\right)<f\left(\vec{RP_{i,1}^{t}}\right) \end{array}\\ \vec{RP_{i,1}^{t}} & else \end{array}\right.$$

In this case, Equation (20) can guide the individual nutcracker to search the area around the reference point RP. If the nutcracker is unable to find quality food at the first reference point, it will then search around the second reference point.

### 3.5. Recovery Strategy

Nutcrackers encounter two situations when searching for their tagged cache locations. The first scenario is that the nutcracker is able to remember exactly where the markers are located. In this case, there are two possibilities: one is that the stored food is still there, and the other is that the food may no longer be there due to weather changes or depredation by other organisms, which is mathematically modeled as shown in Equation (21). ${\vec{X}}_{i,j}^{t+1,new1}$ denotes the position found based on the first reference point.

(21) $${\vec{X}}_{i,j}^{t+1,new1}=\left\{\begin{array}{cc} {\vec{X}}_{i,j}^{t}, & if\;\begin{array}{c} \eta_{6}<\eta_{7} \end{array}\\ {\vec{X}}_{i,j}^{t}+r_{5}\cdot \left({\vec{X}}_{best,j}^{t}-{\vec{X}}_{i,j}^{t}\right)+r_{6}\cdot \left(\vec{RP_{i,1}^{t}}-{\vec{X}}_{C,j}^{t}\right), & else \end{array}\right.$$

where ${\vec{X}}_{C,j}^{t}$ is a randomly selected individual nutcracker from the population. The second case occurs when the nutcrackers cannot remember the position of the first reference point, prompting them to search around the second reference point, which is mathematically modeled as shown in Equation (22).

(22) $${\vec{X}}_{i}^{t+1,2}=\left\{\begin{array}{cc} \vec{X_{i}^{t}}, & if\;\begin{array}{c} f\left(\vec{X_{i}^{t}}\right)<f\left(\vec{RP_{i,2}^{t}}\right) \end{array}\\ \vec{RP_{i,2}^{t}}, & else \end{array}\right.$$

Equation (22) compares the degree of superiority between the reference point and the current position of the nutcracker, selecting the more favorable of these positions. This selection forms the basis for Equation (23), allowing the search to be centered around the chosen superior location. Equation (21) ${\vec{X}}_{i,j}^{t+1,new2}$ denotes the position found based on the second reference point.

(23) $${\vec{X}}_{i,j}^{t+1,new2}=\left\{\begin{array}{cc} {\vec{X}}_{ij}^{t}, & if\;\begin{array}{c} \eta_{8}<\eta_{9} \end{array}\\ {\vec{X}}_{i,j}^{t}+r_{5}\cdot \left({\vec{X}}_{best,j}^{t}-{\vec{X}}_{i,j}^{t}\right)+r_{6}\cdot \left(\vec{RP_{i,2}^{t}}-{\vec{X}}_{C,j}^{t}\right), & else \end{array}\right.$$

The first equation of Equation (23) performs a local search around historical individuals, while the second equation incorporates the current optimal position, the second reference point, and randomly selected individuals from the population to improve global search capability. The overall recovery strategy is presented in Equation (24). The location found during the recovery phase is denoted by $\vec{X_{i}^{t+1(r)}}$.

(24) $$\vec{X_{i}^{t+1(r)}}=\left\{\begin{array}{cc} {\vec{X}}_{i,j}^{t+1,new1}, & if\;\begin{array}{c} \eta_{10}<\eta_{11} \end{array}\\ {\vec{X}}_{i,j}^{t+1,new2}, & else \end{array}\right.$$

To maintain the balance of the NOA algorithm between the selection of reference points, Equation (25) is proposed. $\vec{X_{i}^{t+1(R)}}$ is the optimal location chosen from the first cache point, the second cache point, and the current individual.

(25) $$\vec{X_{i}^{t+1(R)}}=\left\{\begin{array}{cc} {\vec{X}}_{i}^{t+1,1}, & if\;\begin{array}{c} f(Equation\;(20))<f(Equation\;(22)) \end{array}\\ {\vec{X}}_{i}^{t+1,2}, & else \end{array}\right.$$

Equation (26) is designed to maintain a dynamic balance between the caching strategy and the recovery strategy.

(26) $$\vec{X_{i}^{t+1}}=\left\{\begin{array}{cc} \vec{X_{i}^{t+1(r)}}, & if\;\begin{array}{c} \gamma_{2}>P_{a2} \end{array}\\ \vec{X_{i}^{t+1(R)}}, & else \end{array}\right.$$

where $P_{a2}$ is a fixed value of 0.2 and $\gamma_{2}$ is a random number in the interval [0, 1]. The NOA algorithm compares the new individuals with historical individuals in each iteration. If the position of the new individual is closer to the optimal solution, it will replace the historical individual, which is mathematically modeled as shown in Equation (27).

(27) $$\vec{X_{i}^{t+1}}=\left\{\begin{array}{cc} {\vec{X}}_{i}^{t+1}, & if\;\begin{array}{c} f\left({\vec{X}}_{i}^{t+1}\right)<f\left({\vec{X}}_{i}^{t}\right) \end{array}\\ {\vec{X}}_{i}^{t}, & else \end{array}\right.$$

where $f({\vec{X}}_{t}^{t+1})$ denotes the updated fitness value of the *i*-th nutcracker.

## 4. Nutcracker Optimizer Integrated with Hyperbolic Sine–Cosine

The NOA algorithm has several drawbacks, including low convergence accuracy, slow convergence speed, and an inability to fully utilize reference point information. These issues may prevent the NOA algorithm from achieving optimal solutions when addressing complex problems. Therefore, enhancing the performance of NOA algorithms has become a top priority. To address the shortcomings of the NOA, this study introduces a sinh cosh optimizer based on the NOA and proposes a nutcracker optimization algorithm that incorporates hyperbolic sine–cosine.

### 4.1. Hyperbolic Sine–Cosine Optimization Algorithm for Optimal Forage Strategy

In the foraging strategy, individual nutcrackers search randomly throughout the iteration cycle. This strong randomness helps effectively avoid the algorithm getting stuck in local optima; however, it can also result in slower convergence and may even affect the accuracy of the optimization search. For this reason, this paper introduces the SCHO algorithm into the foraging strategy of the NOA algorithm to facilitate searching in the vicinity of the population’s historical optimal positions. In this process, a weight scaling factor $W_{1}$ is introduced. In the initial stages of the iteration, this factor effectively reduces the influence of the historical optimal position on the optimization search process. This enables the algorithm to concentrate more on performing extensive stochastic searches, thereby exploring a larger solution space and avoiding local optima. The role of the weight scaling factor gradually evolves. In the later stages of the algorithm, the focus shifts to searching around the locations of historically optimal individuals, enhancing the efficiency of the nutcracker populations in locating high-quality food sources. This dynamic adjustment mechanism enhances the algorithm’s adaptability and significantly improves the effectiveness of the foraging strategy, enabling it to flexibly respond to search requirements at different stages, which is mathematically modeled as shown in Equation (28).

(28) $$X_{i,j}^{t+1}=X_{i,j}^{t}+r_{7}\times \frac{\sinh r_{8}}{\cosh r_{8}}\left|W_{1}\times X_{\mathrm{best},j}^{t}-X_{i,j}^{t}\right|$$

(29) $$W_{1}=r_{9}\times 2\times \left(-\frac{t}{T}+n\right)$$

where *n* is a sensitivity parameter with a fixed value of 0.5.

### 4.2. Nonlinear Function

In the storage strategy, the third update equation in Equation (13) utilizes a linearly decreasing function to guide the population’s search around the historically optimal positions. Although the linear decreasing function effectively mitigates the rapid loss of diversity in nutcracker populations, it tends to exhibit a relatively slow convergence rate, which may hinder its ability to achieve the desired solution within a limited number of iterations. To tackle this issue, this paper designs a nonlinear function and incorporates it into the food recovery phase of the NOA algorithm. The nonlinear function has a faster rate of change and offers greater flexibility in adjusting the population’s search strategy compared to the linear function *l*. The combination of nonlinear and linear functions, guided by historically optimal individuals, significantly accelerates the food storage process in nutcrackers. Meanwhile, the dynamic adjustment of parameter β diversifies the population’s search direction and reduces the risk of the population becoming stuck in a specific direction for an extended period. This process also improves the convergence speed of the NOA algorithm, which is mathematically represented in Equation (31).

(30) $$\beta =\left\{\begin{array}{cc} 1, & rand<rand\\ -1, & else \end{array}\right.$$

(31) $$\omega =\beta \times r_{14}\times e^{-k\cdot \frac{t}{T}}$$

where *k* is the factor used to control the variation of the nonlinear function, with a value of 250 selected for this paper. The mathematical model developed for the combined use of nonlinear and linear decreasing functions in the storage strategy of the NOA algorithm is presented in Equation (32).

(32) $$\vec{X_{i}^{t+1}}=\left\{\begin{array}{cc} \vec{X_{i}^{t}}+\mu \cdot \left(\vec{X_{best}^{t}}-\vec{X_{i}^{t}}\right)\cdot \left|\lambda \right|+r_{2}\cdot \left(\vec{X_{A}^{t}}-\vec{X_{B}^{t}}\right), & if\;\begin{array}{c} \eta_{1}<\eta_{2} \end{array}\\ \vec{X_{best}^{t}}+\mu \cdot \left(\vec{X_{A1}^{t}}-\vec{X_{B1}^{t}}\right), & if\;\begin{array}{c} \eta_{1}<\eta_{5} \end{array}\\ \omega \cdot \vec{X_{best}^{t}}\cdot l, & Otherwise \end{array}\right.$$

### 4.3. Hyperbolic Sine–Cosine Optimization Algorithm for Optimal Recovery Strategy

During the food recovery phase, the nutcracker population searches around reference points, random individuals, and the current optimal individual. Due to the nutcracker’s limitations in accurately remembering the locations of reference points and random individuals, the NOA algorithm may not fully leverage the location information of the reference point, which can impact the overall recovery strategy. This situation can result in nutcrackers searching aimlessly within the area, potentially preventing them from locating stored food. In contrast, the exploration process of the SCHO algorithm targets a specific region for searching, allowing it to effectively integrate the information from reference points, random individuals, and the current optimal individual?s position. This integrated information is then used as the primary reference point for further searches. However, the SCHO algorithm’s iterative approach, which focuses on updating around the current optimal individual, can reduce population diversity and expose the algorithm to local optima. Therefore, to enhance the algorithm’s performance, an improved update formula for SCHO is proposed to ensure that the nutcracker population can fully utilize the integrated reference point information, as shown in Equation (33).

(33) $$X_{i,j}^{t+1}=\left\{\begin{array}{cc} X_{i,j}^{t}+r_{10}\times W_{2}\times X_{best,j}^{t}, & r_{11}>0.5\\ X_{i,j}^{t}-r_{10}\times W_{2}\times X_{best,j}^{t}, & r_{11}<0.5 \end{array}\right.$$

(34) $$W_{2}=r_{12}\times a_{1}\times \left(\cosh r_{13}+u\times \sinh r_{13}-1\right)$$

(35) $$a_{1}=3\times \left(-1.3\times \frac{t}{T}+m\right)$$

In Equations (33)–(35), $W_{2}$ serves as a weighting factor that scales the degree of utilization of historically optimal individuals in the search for the ideal solution, and *m* and μ are fixed at values of 0.45 and 0.388, respectively.In summary, we can obtain the pseudo-code of ISCHNOA as in Algorithm 1.
**Algorithm 1 The ISCHNOA**Input: population size *N*, the lower limits of variables $u_{b}$, the upper limits of variables $u_{l}$,the current number of iteration *t* = 0, and the maximum number of iteration *T*Output: the best solution1. Initialize N nutcracker using Equation (10)2. Identifying the optimal individual based on fitness values.3.    While (*t* < *T*)4.       Generate random numbers $\sigma_{1}$ and $\sigma_{2}$ between 0 and 1.5.       If $\sigma_{1}$ < $\sigma_{2}$6.          ϕ is a random number between 0 and 1.7.          for i = 1:N8.             for j = i:d9.             if $\phi >P_{a1}$10.                Updating ${\vec{X}}_{i}^{t+1}$ using Equation (11), (27) and (28).11.             else12.                Updating ${\vec{X}}_{i}^{t+1}$ using Equations (27) and (32).13.             end if14.          end for15.          t = t + 116.       end for17.    Else18.       The reference point matrix is generated using Equations (15)–(17).19.       Generate random numbers φ between 0 and 1.20.       for i = 1:N21.          if $\varphi >P_{a2}$22.             Updating ${\vec{X}}_{i}^{t+1}$ using Equations (24), (27) and (33).23.          else24.             Updating ${\vec{X}}_{i}^{t+1}$ using Equations (25), (27) and (33).25.          end if26.          t = t + 127.       end for28.    end while

## 5. Simulation Experiments and Result Analysis

All simulations were performed in Matlab 2018. Experimental equipment included a computer with an Intel Core i7-9750H 2.60GHz CPU, GTX 1660Ti GPU, and Windows 10 64-bit operating system.

### 5.1. Introduction to Test Functions and Parameter Settings

To verify the effectiveness of the ISCHNOA algorithm, this paper first employs classical benchmark functions (Table 1) to evaluate the performance of the improved NOA in solving complex problems. Classical benchmark test functions can be divided into unimodal, multimodal, and composite categories. The unimodal test function contains a single optimal value and is primarily used to evaluate the local search capabilities and convergence speed of optimization algorithm. The multimodal test function, in contrast, features multiple extrema and is primarily used to assess an algorithm’s exploration capabilities. It evaluates the algorithm’s ability to locate a globally optimal solution within a search space that contains both a global optimum and several local optima. The composite benchmarking function is used to assess the algorithm’s ability to handle various types of problems and test its capacity to maintain a dynamic balance between global and local search. Subsequently, this paper employs the CEC2014 (Table 2) and CEC2020 (Table 3) test suites to further validate the performance of the ISCHNOA algorithm. The functions in these two test suites are more complex than classical benchmark functions, imposing higher demands on the performance of the solution algorithms. To further validate the effectiveness of the improvements, this paper compares the ISCHNOA algorithm with several recently published optimization algorithms, including the Honey Badger Algorithm [47] (HBA), the Equilibrium Optimizer [48] (EO), Sinh Cosh optimizer, and nutcracker optimizer. It is also compared with some highly cited algorithms, such as the Whale Optimization Algorithm [49] (WOA) and the Grey Wolf Optimizer [50] (GWO). The optimization algorithms mentioned above are designed for solving mathematical optimization problems, and the parameters used are set according to the authors’ recommendations in the literature. In this paper, we set the population size *N* to 25, the number of iterations for the classical benchmark test functions to 500, and the number of evaluations for the CEC2014 and CEC2020 test suites to 50,000. $P_{a1}$, $P_{a2}$, and δ are the three main control parameters of the NOA algorithm, which are fixed at 0.2, 0.4, and 0.05 in the ISCHNOA algorithm to ensure a fair comparison of the algorithms.

### 5.2. Classical Benchmark Function

**Figure 4:**
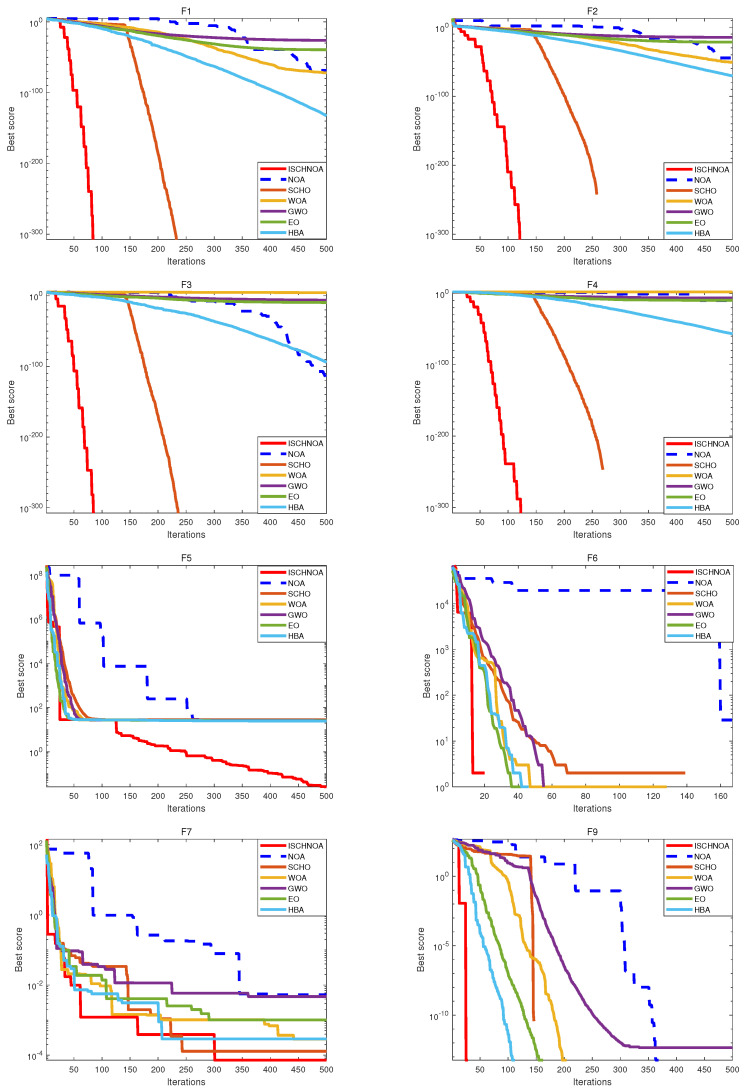
Convergence curves of each algorithm for the test function.

**Figure 5:**
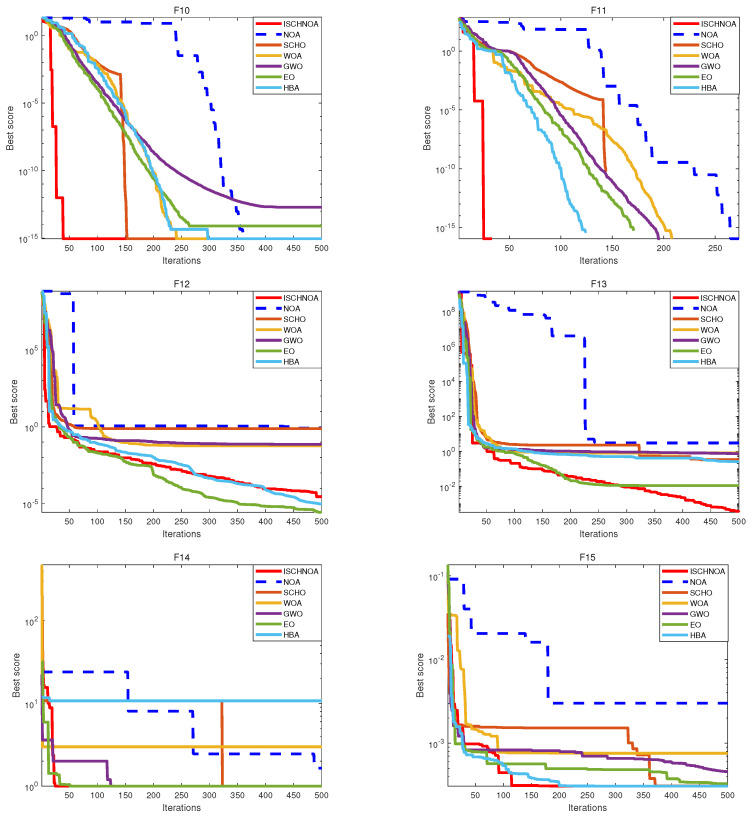
Convergence curves of each algorithm for the test function (continued).

In this paper, the ISCHNOA algorithm is evaluated for its exploration capabilities and its effectiveness in escaping local optima through tests on 14 classical benchmark functions. Based on Figure 4 and Figure 5, it can be concluded that while the ISCHNOA algorithm may not achieve the same level of convergence accuracy as some of the comparison algorithms for certain test functions, it exhibits the fastest convergence speed among them. This indicates that the ISCHNOA algorithm offers a significant advantage in quickly identifying near-optimal solutions, making it particularly well-suited for optimization problems that demand rapid responses. Overall, the improved algorithm demonstrates strong performance and considerable potential.

As illustrated in Figure 4 and Figure 5, the ISCHNOA algorithm demonstrates superior performance compared to the comparison algorithm for all functions in the unimodal problem—except for F6, where its accuracy is slightly lower. Furthermore, ISCHNOA converges twice as quickly as the other algorithms. In multimodal functions F9∼F13, and the fixed dimension multimodal functions F14 and F15, the ISCHNOA algorithm exhibits a reduced tendency to be influenced by local optimal solutions when compared to the benchmark algorithm, while maintaining the fastest convergence speed. This further underscores the effectiveness of the proposed improvement strategy in enhancing both exploration capability and convergence speed. The results indicate that the ISCHNOA algorithm demonstrates remarkable local search capability, can effectively escape from local optima in a timely manner, and exhibits a rapid convergence speed.

### 5.3. Comparison of Algorithms in the CEC2014 Suite

The ISCHNOA algorithm is analyzed in depth using the CEC2014 test suite to verify the effectiveness of the proposed improvement strategy. The CEC2014 suite of functions is more varied and allows for a detailed exploration of the algorithms’ performance in several aspects, including their exploitation capabilities, exploration abilities, and their capacity to escape from local optima. The dimension selected for the CEC2014 test suite in this paper is 10.

Based on the mean, variance, and ranking achieved by the algorithms listed in Table 4, it can be inferred that the ISCHNOA algorithm is the best overall. The ISCHNOA algorithm consistently ranks first among the other functions, except for functions F32, F33, F41, and F46, where its performance is slightly lower than that of the NOA algorithm. In the CEC2014 test suite, the NOA algorithm was ranked second overall, while the last-ranked algorithm was HBA. Benchmark information for the thirty test functions in the CEC2014 suite is provided in Table 2.

### 5.4. Comparison of Algorithms in the CEC2020 Suite

The more representative CEC2020 test suite was chosen to validate the performance of the improved algorithm, with the dimension set to 10. The test suite contains ten functions, ranging from simple to complex. These include single-peak functions (F54), multi-peak functions (F55∼F57), hybrid functions (F58∼F60), and combined functions (F61∼F63). Table 5 presents the mean, standard deviation, and algorithm rankings following 30 independent runs. The ISCHNOA algorithm achieves relative optimization in all but F58 of the ten tested functions. Additionally, only two functions show slightly higher standard deviations compared to the NOA algorithm; however, this does not impact the overall ranking of the ISCHNOA algorithm among the compared algorithms.

### 5.5. UAV Path Planning in Complex Environment

The convergence of a UAV path planning problem typically refers to the ability of the algorithm to successfully find an optimal or near-optimal path within a given time frame or a specified number of iterations, ultimately reaching a steady state under specific conditions. In the UAV path planning problem, both convergence speed and convergence accuracy are crucial factors. Convergence speed determines whether a viable path can be found within an acceptable timeframe during emergencies, while convergence accuracy influences the cost required for the UAV to reach its target and even impacts flight safety. In this paper, the ISCHNOA algorithm is applied to the UAV path optimization problem in complex environments to assess its effectiveness regarding convergence speed and convergence accuracy. Comparative experiments are carried out to analyze the performance of the ISCHNOA algorithm across various scenarios and evaluate its feasibility for practical applications. The results show that the ISCHNOA algorithm can quickly identify near-optimal paths while reducing flight costs and enhancing the operational efficiency of UAVs in complex environments, all while ensuring safety. This provides robust support for the use of drones in real-world operations.

#### 5.5.1. Setting the Scene

In this paper, a digital elevation model of Christmas Island, Australia, was created using LiDAR sensors [51]. Eight obstacles, each representing a different level of difficulty, were established in two distinct areas for UAV path planning tests. By comparing ISCHNOA with NOA, WOA, GWO, HBA, EO, and SCHO applied to simulated maps, the performance of each algorithm for path planning in complex environments is evaluated [52]. Table 6 details the key parameters of the UAV path planning experiments to ensure that the simulations are realistic and reliable, providing a solid reference for practical applications. The comparative results further validate the advantages of the ISCHNOA algorithm in handling complex environments. The information about the obstacles and the cost obtained by each algorithm is displayed in Table 7.

#### 5.5.2. Analysis of the UAV Path Planning Results

**Figure 6:**
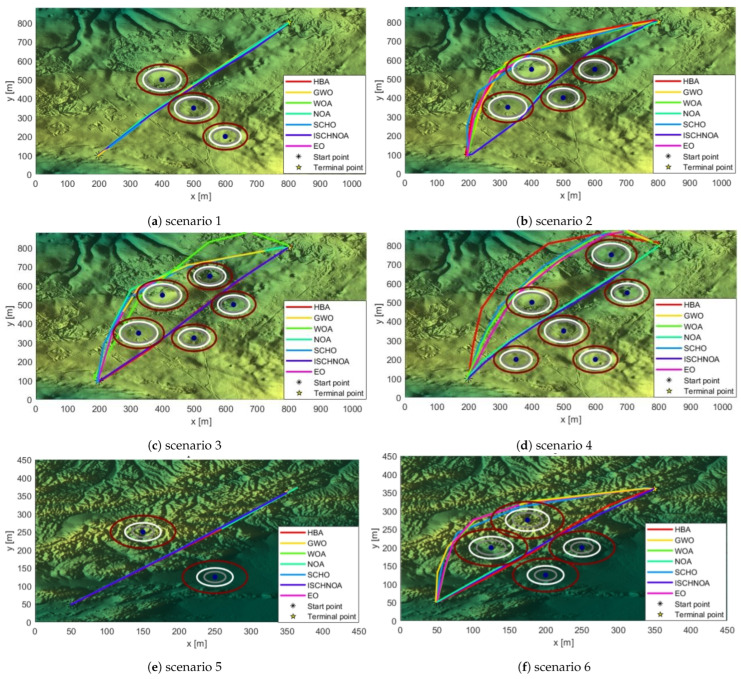

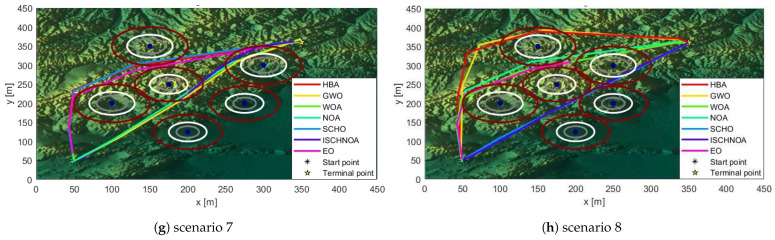
Top view of each algorithm’s result across eight different obstacle classes.

Figure 6 illustrates the top view of UAV path planning using the ISCHNOA algorithm across eight distinct obstacle classes. It effectively showcases the planned paths of the UAV and highlights their safety under varying obstacle densities. Comparing different levels of obstacles reveals noticeable changes in path selection. Notably, all algorithms are able to identify a relatively safe path, irrespective of the obstacle density. However, the optimal paths identified by these algorithms across the eight different obstacle class maps vary significantly due to the differences in their respective performance. The dark blue dots and gray circles indicate the collision areas. Since UAVs have physical dimensions, the area between the gray boundary and the white boundary is defined as the threat area, while the region beyond the white boundary is considered safe. This distinction aids in better assessing the effectiveness of path planning and its safety.

#### 5.5.3. Total Cost of the Drone Path

**Figure 7:**
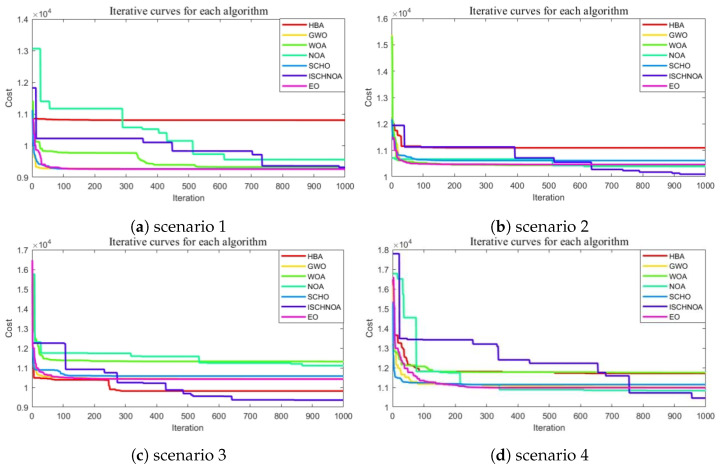

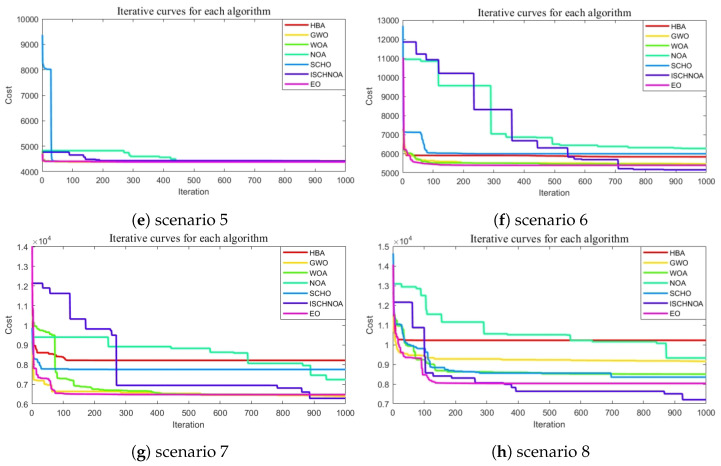
Convergence plots of each algorithm for the eight obstacle class cases.

As can be seen from the planned roadmap, the optimal paths identified by the algorithm vary significantly across different scenarios. Since the UAV path cost must account for multiple factors, it is essential to utilize the fitness value graph to represent the total cost of the path effectively. Figure 7 illustrates the convergence trend of the optimal fitness values for the seven top-performing algorithms throughout the iteration process. It is evident that the algorithms identify similar optimal paths with relatively comparable total costs when faced with sparse obstacles. However, the advantages of the ISCHNOA algorithm become increasingly evident in complex scenarios and under high obstacle density conditions. Compared to the NOA algorithm, the ISCHNOA algorithm effectively avoids getting trapped in local optimal solutions. This further validates the effectiveness of the improvement strategy proposed in this paper.

The information about the obstacles and the cost obtained by each algorithm is displayed in Table 7.

#### 5.5.4. Sensitivity Analysis

Meanwhile, the adaptability of the ISCHNOA algorithm is further evaluated by adjusting the parameter *k* within a specified range. As shown in Table 8, *k* varies in the interval [150, 350]. In both the simple and obstacle-dense scenarios, changes in *k* typically result in only a small fluctuation in the cost.

## 6. Conclusions and Outlook

This paper presents an improved nutcracker optimization algorithm that integrates hyperbolic positive cosine (ISCHNOA) to enhance its effectiveness in tackling complex optimization problems and UAV path planning. Integrating the SCHO algorithm into the NOA algorithm enhances its exploration capability, enabling the UAV to find a flight path with lower cost and higher safety. Additionally, a nonlinear function is introduced to further accelerate the algorithm’s convergence speed, allowing the UAV to identify a suitable flight path in less time. The ISCHNOA algorithm’s capability to explore and escape from locally optimal solutions was assessed using standard classical test functions, as well as the CEC2014 and CEC2020 test suites. Meanwhile, this paper presents a UAV path planning model that incorporates distance cost, threat source cost, altitude cost, and path smoothing cost. The ISCHNOA algorithm is applied to identify suitable routes for UAVs across various complex scenarios. Experimental results indicate that the proposed ISCHNOA algorithm demonstrates strong performance across a variety of numerical optimization problems. The algorithm typically achieves near-optimal solutions in low-dimensional cases; however, the performance of the ISCHNOA algorithm is less remarkable on high-dimensional problems. This presents an opportunity for further research in the future. Future research will concentrate on optimizing the ISCHNOA algorithm to enhance its performance on high-dimensional problems, allowing it to better address challenges in various complex scenarios. Additionally, we plan to apply the ISCHNOA algorithm to a range of UAV tasks in urban environments.

## Figures and Tables

**Table 1:** Description of some standard test functions.

Function	Dimension	Value	Globally Optimal
$f_{1}\left(x\right)=\sum_{i=1}^{n}x_{i}^{2}$	30	[−100, 100]	0
$f_{2}\left(x\right)=\sum_{i=1}^{n}\left|x_{i}\right|+\prod_{i=1}^{n}\left|x_{i}\right|$	30	[−10, 10]	0
$f_{3}\left(x\right)=\sum_{i=1}^{n}{\left(\sum_{j=1}^{i}x_{j}\right)}^{2}$	30	[−10, 10]	0
$f_{4}\left(x\right)=\max_{i}\left\{\left|x_{i}\right|,1\leq i\leq n\right\}$	30	[−10, 10]	0
$f_{5}\left(x\right)=\sum_{i=1}^{n-1}\left[100{\left(x_{i+1}-x_{i}^{2}\right)}^{2}+{\left(x_{i}-1\right)}^{2}\right]$	30	[−10, 10]	0
$f_{6}\left(x\right)=\sum_{i=1}^{n}{\left(\left[x_{i}+0.5\right]\right)}^{2}$	30	[−10, 10]	0
$f_{7}\left(x\right)=\sum_{i=1}^{n}ix_{i}^{4}+randm\left[0,1\right)$	30	[−10, 10]	0
$f_{8}\left(x\right)=\sum_{i=1}^{n}\left[x_{i}^{2}-10\cdot \cos\left(2\pi x_{i}\right)+10\right]$	30	[−5.12, 5.12]	0
$f_{9}\left(x\right)=-20\exp\left(-0.2\cdot \sqrt{\frac{1}{n}\sum_{i=1}^{n}x_{i}^{2}}\right)-\exp\left(\frac{1}{n}\sum_{i=1}^{n}\cos\left(2\pi x_{i}\right)\right)+20+e$	30	[−32, 32]	0
$f_{10}\left(x\right)=\frac{1}{4000}\sum_{i=1}^{n}x_{i}^{2}-\prod_{i=1}^{n}\cos\left(\frac{x_{i}}{\sqrt{i}}\right)+1$	30	[−600, 600]	0
$\begin{array}{c} f_{11}\left(x\right)=\frac{\pi }{n}\left\{10\cdot \sin^{2}\left(\pi y_{i}\right)+\sum_{i=1}^{n-1}{\left(y_{i}-1\right)}^{2}\right.\left[1+10\cdot \sin^{2}\left(\pi y_{i+1}\right)\right]\\ +\left.{\left(y_{n}-1\right)}^{2}\right\}+\sum_{i=1}^{n}u(x_{i},10,100,4),\begin{array}{cc} & y_{i}=1+\frac{1}{4}\left(x_{i}+1\right) \end{array}\\ \begin{array}{c} u\left(x_{i},a,k,m\right)=\left\{\begin{array}{cc} k\cdot {\left(x_{i}-a\right)}^{m}, & x_{i}>a\\ 0, & -a\leq x_{i}\leq a\\ k\cdot {\left(-x_{i}-a\right)}^{m}, & x_{i}<-a \end{array}\right. \end{array} \end{array}$	30	[−50, 50]	0
$\begin{array}{c} f_{12}\left(x\right)=0.1\cdot \left\{\sin^{2}\left(3\pi x_{1}\right)+\sum_{i=1}^{n-1}{\left(x_{i}-1\right)}^{2}\right.\left[1+\sin^{2}\left(3\pi x_{i+1}\right)\right]\\ \begin{array}{cc} & \left.+{\left(x_{n}-1\right)}^{2}\left[1+\sin^{2}\left(2\pi x_{n}\right)\right]\right\} \end{array}+\sum_{i=1}^{n}u(x_{i},5,100,4) \end{array}$	30	[−50, 50]	0
$f_{13}=\left[\frac{1}{500}+\sum_{j=1}^{25}\frac{1}{j+\sum_{i=1}^{2}{\left(x_{i}-a_{ij}\right)}^{6}}\right]$	2	[−65, 65]	1
$f_{14}\left(x\right)=\sum_{i=1}^{11}{\left[a_{i}-\frac{x_{1}\left(b_{i}^{2}+b_{i}x_{2}\right)}{{b_{i}}^{2}+b_{i}x_{3}+x_{4}}\right]}^{2}$	4	[−5, 5]	0.0003075

**Table 2:** Characteristics of CEC-2014 test functions.

Class	ID	Function	Globally Optimal
Unimodal	F24	Function of Rotated High Conditioned Elliptic	100
function	F25	Function of Rotated Bent Cigar	300
	F26	Function of Rotated Discus	
Simple	F27	Function of Shifted and Rotated Rosenbrock	400
multimodal	F28	Function of Shifted and Rotated Ackley	500
Test function	F29	Function of Shifted and Rotated Weierstrass	600
	F30	Function of Shifted and Rotated Griewank	700
	F31	Function of Shifted Rastrigin	800
	F32	Function of Shifted and Rotated Rastrigin	900
	F33	Function of Shifted Schwefel	1000
	F34	Function of Shifted and Rotated Schwefel	1100
	F35	Function of Shifted and Rotated Katsuura	1200
	F36	Function of Shifted and Rotated HappyCat	1300
	F37	Function of Shifted and Rotated HGBat	1400
	F38	Function of Shifted and Rotated Expanded Griewank’s	
		plus Rosenbrock	1500
	F39	Function of Shifted and Rotated Expanded Scaffer	1600
Hybrid test	F40	Function 1 Hybrid	1700
Functions	F41	Function 2 Hybrid	1800
	F42	Function 3 Hybrid	1900
	F43	Function 4 Hybrid	2000
	F44	Function 5 Hybrid	2100
	F45	Function 6 Hybrid	2200
Composition	F46	Function 1 Composition	2300
test	F47	Function 2 Composition	2400
Functions	F48	Function 3 Composition	2500
	F49	Function 4 Composition	2600
	F50	Function 5 Composition	2700
	F51	Function 6 Composition	2800
	F52	Function 7 Composition	2900
	F53	Function 8 Composition	3000

**Table 3:** Characteristics of CEC-2020 test functions.

Class	ID	Function	Globally Optimal
Unimodal function	F54	Shifted and Rotated Bent Cigar function	100
Basic function	F55	Shifted and Rotated Schwefel’s function	1100
	F56	Shifted and Rotated Lunacek bi-Rastrigin function	700
	F57	Expanded Rosenbrock’s plus Griewangk’s function	1900
Hybrid Function	F58	Hybrid Function 1 (N = 3)	1700
	F59	Hybrid Function 2 (N = 4)	1600
	F60	Hybrid Function 3 (N = 5)	2100
Composition Function	F61	Composition Function 1 (N = 3)	2200
	F62	Composition Function 2 (N = 4)	2400
	F63	Composition Function 3 (N = 5)	2500

**Table 4:** Mean, variance, and ranking achieved by each algorithm on the CEC2014 suite.

F	Index	ISCHNOA	NOA	SCHO	WOA	GWO	HBA	EO
Unimodal								
F24	Ave	100	100	$7.85\times 10^{5}$	$4.95\times 10^{6}$	$6.05\times 10^{6}$	$1.12\times 10^{7}$	$5.24\times 10^{4}$
	std	$5.33\times 10^{-4}$	$1.37\times 10^{-4}$	$9.68\times 10^{6}$	$4.65\times 10^{6}$	$5.62\times 10^{6}$	$5.76\times 10^{6}$	$6.65\times 10^{4}$
	Rank	2	1	4	5	6	7	3
F25	Ave	200.00	200	$2.84\times 10^{6}$	$8.41\times 10^{5}$	$1.73\times 10^{7}$	$1.28\times 10^{7}$	$1.43\times 10^{3}$
	std	$3.6\times 10^{-6}$	$3.07\times 10^{-9}$	$4.85\times 10^{6}$	$6.03\times 10^{5}$	$6.59\times 10^{7}$	$5.40\times 10^{7}$	$1.44\times 10^{3}$
	Rank	2	1	5	4	7	6	3
F26	Ave	300	300	$5.95\times 10^{3}$	$4.92\times 10^{4}$	$5.87\times 10^{3}$	$1.06\times 10^{4}$	$4.39\times 10^{2}$
	std	$1.19\times 10^{-9}$	$2.7\times 10^{-11}$	$3.21\times 10^{3}$	$2.91\times 10^{4}$	$3.95\times 10^{3}$	$4.16\times 10^{3}$	$2.03\times 10^{2}$
	Rank	2	1	5	7	4	6	3
Multimodal								
F27	Ave	411.00	418.69	429.52	$4.41\times 10^{2}$	$4.30\times 10^{2}$	$5.70\times 10^{2}$	$4.28\times 10^{2}$
	std	16.2939	17.0336	28.305	$2.78\times 10^{1}$	$2.04\times 10^{1}$	$5.05\times 10^{1}$	$2.41\times 10^{1}$
	Rank	1	2	4	6	5	7	3
F28	Ave	$5.18\times 10^{2}$	$5.20\times 10^{2}$	$5.20\times 10^{2}$	$5.20\times 10^{2}$	$5.20\times 10^{2}$	$5.21\times 10^{2}$	$5.20\times 10^{2}$
	std	6.3445	$2.17\times 10^{-2}$	$1.09\times 10^{-1}$	$1.14\times 10^{-1}$	$1.04\times 10^{-1}$	$1.75\times 10^{-1}$	$6.74\times 10^{-2}$
	Rank	1	2	3	5	4	6	4
F29	Ave	600	600	$6.02\times 10^{2}$	$6.08\times 10^{2}$	$6.02\times 10^{2}$	$6.23\times 10^{2}$	$6.02\times 10^{2}$
	std	$5.33\times 10^{-4}$	$8.08\times 10^{-4}$	1.49	1.54	1.52	$4.72\times 10^{2}$	1.52
	Rank	1	2	3	5	4	6	4
F30	Ave	700	700	$7.02\times 10^{2}$	$7.01\times 10^{2}$	$7.02\times 10^{2}$	$7.01\times 10^{2}$	700
	std	$2.55\times 10^{-2}$	$2.13\times 10^{-2}$	1.22	$4.52\times 10^{-1}$	2.78	$2.84\times 10^{-1}$	$3.32\times 10^{-2}$
	Rank	3	2	6	5	7	4	1
F31	Ave	800	800	$8.05\times 10^{2}$	$8.38\times 10^{2}$	$8.08\times 10^{2}$	$8.92\times 10^{2}$	$8.08\times 10^{2}$
	std	$1.5\times 10^{-11}$	$1.0\times 10^{-12}$	1.10	$1.43\times 10^{1}$	3.23	$2.38\times 10^{1}$	2.89
	Rank	2	1	3	6	5	7	4
F32	Ave	906.00	903.28	$9.18\times 10^{2}$	$9.46\times 10^{2}$	$9.15\times 10^{2}$	$9.84\times 10^{2}$	$9.11\times 10^{2}$
	std	3.56	1.10	$1.01\times 10^{1}$	$1.48\times 10^{1}$	7.15	$2.99\times 10^{1}$	5.11
	Rank	2	1	5	6	4	7	3
F33	Ave	$1.00\times 10^{3}$	$1.00\times 10^{3}$	$1.12\times 10^{3}$	$1.52\times 10^{3}$	$1.31\times 10^{3}$	$2.81\times 10^{3}$	$1.18\times 10^{3}$
	std	1.604	2.2748	$1.58\times 10^{1}$	$2.49\times 10^{2}$	$1.89\times 10^{2}$	$1.25\times 10^{2}$	$1.15\times 10^{2}$
	Rank	1	2	3	6	5	7	4
F34	Ave	1332.00	1523.04	$1.56\times 10^{3}$	$2.91\times 10^{3}$	$1.84\times 10^{3}$	$1.99\times 10^{3}$	$1.70\times 10^{3}$
	std	182.78	190.36	$1.51\times 10^{2}$	$5.11\times 10^{2}$	$3.83\times 10^{2}$	$4.29\times 10^{2}$	$3.34\times 10^{2}$
	Rank	1	2	3	7	5	6	4
F35	Ave	1200.00	1200.204	1200.08	1200.79	1200.81	1200.57	1200.33
	std	$7.58\times 10^{-2}$	$3.5\times 10^{-2}$	$1.81\times 10^{-2}$	$2.71\times 10^{-1}$	$6.53\times 10^{-1}$	$3.64\times 10^{-1}$	$2.11\times 10^{-1}$
	Rank	1	3	2	6	7	5	4
F36	Ave	1300.00	1300.109	1300.05	1300.34	1300.19	1300.20	1300.06
	std	$2.84\times 10^{-2}$	$3.4\times 10^{-2}$	$1.29\times 10^{-3}$	$1.29\times 10^{-1}$	$5.53\times 10^{-2}$	$9.69\times 10^{-2}$	$4.05\times 10^{-3}$
	Rank	1	4	2	7	5	6	3
F37	Ave	1400.00	1400.15	1400.07	1400.30	1400.32	1400.25	1400.17
	std	$3.19\times 10^{-2}$	$5.4\times 10^{-2}$	$9.91\times 10^{-2}$	$2.21\times 10^{-1}$	$2.24\times 10^{-1}$	$2.04\times 10^{-1}$	$7.64\times 10^{-2}$
	Rank	1	3	2	6	7	5	4
F38	Ave	1500.00	1500.75	1501.75	1506.98	1501.55	1500.57	1501.12
	std	0.29	0.21	1.13	2.32	$7.48\times 10^{-1}$	$5.89\times 10^{-1}$	$5.99\times 10^{-1}$
	Rank	1	3	6	7	5	2	4
F39	Ave	1602.00	1602.22	1603.32	1603.39	1602.61	1600.98	1602.23
	std	0.31	0.19	$5.09\times 10^{-1}$	$3.12\times 10^{-1}$	$7.46\times 10^{-1}$	$3.51\times 10^{-1}$	$4.67\times 10^{-1}$
	Rank	2	3	7	6	5	1	4
Hybrid								
F40	Ave	1715.63	1717.13	$1.81\times 10^{4}$	$2.03\times 10^{5}$	$4.08\times 10^{4}$	$9.16\times 10^{3}$	$4.36\times 10^{3}$
	std	9.54	5.55	$4.86\times 10^{3}$	$3.86\times 10^{5}$	$1.05\times 10^{5}$	$1.04\times 10^{4}$	$2.26\times 10^{3}$
	Rank	1	2	5	7	6	4	3
F41	Ave	1801.00	1800.89	$7.95\times 10^{3}$	$1.19\times 10^{4}$	$1.23\times 10^{4}$	$9.75\times 10^{3}$	$8.74\times 10^{3}$
	std	0.47	0.60	$6.59\times 10^{3}$	$1.18\times 10^{4}$	$7.67\times 10^{3}$	$7.50\times 10^{3}$	$5.63\times 10^{3}$
	Rank	2	1	3	6	7	5	4
F42	Ave	1900.00	1900.31	1901.37	1905.94	1902.75	1903.55	1901.52
	std	0.32	0.14	$9.12\times 10^{-1}$	1.37	1.12	1.68	$8.04\times 10^{-1}$
	Rank	1	2	3	7	5	6	4
F43	Ave	2000.00	2000.70	$4.11\times 10^{3}$	$8.11\times 10^{3}$	$7.27\times 10^{3}$	$3.41\times 10^{3}$	$2.13\times 10^{3}$
	std	0.45	0.61	$2.76\times 10^{2}$	$4.26\times 10^{3}$	$4.46\times 10^{3}$	$1.87\times 10^{3}$	$6.32\times 10^{1}$
	Rank	1	2	5	7	6	4	3
F44	Ave	2100.00	2100.70	$3.63\times 10^{3}$	$8.87\times 10^{4}$	$9.63\times 10^{3}$	$2.33\times 10^{3}$	$2.40\times 10^{3}$
	std	0.37	0.26	$3.26\times 10^{2}$	$4.26\times 10^{3}$	$4.37\times 10^{3}$	$4.51\times 10^{3}$	$1.96\times 10^{2}$
	Rank	1	2	5	7	6	3	4
F45	Ave	2200.00	2200.27	$2.27\times 10^{3}$	$2.29\times 10^{3}$	$2.30\times 10^{3}$	$2.21\times 10^{3}$	$2.24\times 10^{3}$
	std	0.37	0.18	$6.29\times 10^{1}$	$7.29\times 10^{1}$	$5.77\times 10^{1}$	$6.89\times 10^{1}$	$3.89\times 10^{1}$
	Rank	1	2	5	6	7	3	4
Composition								
F46	Ave	2500	2500	$2.53\times 10^{3}$	$2.62\times 10^{3}$	$2.63\times 10^{3}$	$2.51\times 10^{3}$	$2.63\times 10^{3}$
	std	0	0	$2.61\times 10^{1}$	$4.06\times 10^{1}$	4.02	$2.26\times 10^{1}$	$2.9\times 10^{-10}$
	Rank	1	2	4	5	7	3	6
F47	Ave	$2.55\times 10^{3}$	$2.51\times 10^{3}$	$2.52\times 10^{3}$	$2.58\times 10^{3}$	$2.54\times 10^{3}$	$2.56\times 10^{3}$	$2.55\times 10^{3}$
	std	43.14	5.38	26.1	31.4	34.6	35.3	35.0
	Rank	5	1	2	7	3	6	4
F48	Ave	2622.00	2624.41	2640.07	$2.69\times 10^{3}$	$2.70\times 10^{3}$	$2.68\times 10^{3}$	$2.70\times 10^{3}$
	std	14.03	27.30	10.9	9.32	12.9	27.0	14.4
	Rank	1	2	3	4	6	5	7
F49	Ave	2700.00	2700.10	2700.25	2700.38	2700.14	2700.02	2700.07
	std	$2.08\times 10^{-2}$	$2.00\times 10^{-2}$	$3.31\times 10^{-1}$	$1.72\times 10^{-1}$	$1.82\times 10^{1}$	$1.17\times 10^{-1}$	$3.37\times 10^{-2}$
	Rank	1	4	6	7	5	2	3
F50	Ave	2781.00	2820.83	$2.80\times 10^{3}$	$3.10\times 10^{3}$	$2.98\times 10^{3}$	$2.95\times 10^{3}$	$3.02\times 10^{3}$
	std	$1.01\times 10^{2}$	$1.02\times 10^{2}$	$7.21\times 10^{2}$	$1.13\times 10^{2}$	$1.46\times 10^{2}$	$9.06\times 10^{1}$	$1.12\times 10^{2}$
	Rank	1	3	2	7	4	5	6
F51	Ave	3000	3000	3000	$3.38\times 10^{3}$	$3.27\times 10^{3}$	$3.19\times 10^{3}$	$3.22\times 10^{3}$
	std	0	0	0	$1.48\times 10^{2}$	$7.62\times 10^{1}$	$1.26\times 10^{2}$	$5.14\times 10^{1}$
	Rank	1	1	1	5	4	2	3
F52	Ave	3100.00	3101.27	3100.00	$1.77\times 10^{5}$	$3.77\times 10^{5}$	$3.45\times 10^{5}$	$2.41\times 10^{5}$
	std	0	34.75	$1.9\times 10^{2}$	$5.26\times 10^{5}$	$8.60\times 10^{5}$	$1.28\times 10^{5}$	$6.18\times 10^{5}$
	Rank	1	3	2	4	7	6	5
F53	Ave	3200.00	3451.85	3200.00	$5.18\times 10^{3}$	$4.45\times 10^{3}$	$3.47\times 10^{3}$	$3.74\times 10^{3}$
	std	0	60.78	61.82	$1.19\times 10^{3}$	$7.85\times 10^{2}$	$6.75\times 10^{2}$	$2.94\times 10^{2}$
	Rank	1	3	2	7	6	4	5
	Ave Rank	1.43	2.10	3.77	6.03	4.66	4.76	3.83

**Table 5:** Mean, variance, and ranking achieved by each algorithm on the CEC2020 suite.

F	Index	ISCHNOA	NOA	SCHO	WOA	GWO	HBA	EO
F54	Ave	100	100	$4.80\times 10^{8}$	$3.05\times 10^{6}$	$4.89\times 10^{7}$	$1.88\times 10^{2}$	$3.19\times 10^{3}$
	std	$7.33\times 10^{-5}$	$2.17\times 10^{-9}$	$3.72\times 10^{8}$	$7.30\times 10^{6}$	$1.40\times 10^{8}$	$1.55\times 10^{3}$	$2.86\times 10^{3}$
	Rank	2	1	7	5	6	3	4
F55	Ave	1219.40	$1.23\times 10^{3}$	$1.59\times 10^{3}$	$2.12\times 10^{3}$	$1.54\times 10^{3}$	$1.47\times 10^{3}$	$1.52\times 10^{3}$
	std	82.82	$9.56\times 10^{1}$	$2.47\times 10^{2}$	$3.39\times 10^{2}$	$1.94\times 10^{2}$	$1.87\times 10^{2}$	$2.11\times 10^{2}$
	Rank	1	2	6	7	5	3	4
F56	Ave	$7.16\times 10^{2}$	$7.16\times 10^{2}$	$7.51\times 10^{2}$	$7.78\times 10^{2}$	$7.30\times 10^{2}$	$7.25\times 10^{2}$	$7.21\times 10^{2}$
	std	2.31	2.84	$1.8\times 10^{1}$	$2.11\times 10^{1}$	8.20	$1.46\times 10^{1}$	6.72
	Rank	1	2	6	7	5	4	3
F57	Ave	1900.93	1900.00	$2.46\times 10^{3}$	1900.05	1900.37	1900.00	1900.00
	std	0.21	0	$2.66\times 10^{3}$	$1.99\times 10^{-1}$	$5.44\times 10^{-1}$	0	0
	Rank	4	1	5	2	3	1	1
F58	Ave	1717.56	1716.81	$5.98\times 10^{4}$	$3.40\times 10^{5}$	$5.29\times 10^{4}$	$2.87\times 10^{3}$	$3.90\times 10^{3}$
	std	11.45	10.85	$1.43\times 10^{5}$	$6.45\times 10^{5}$	$1.22\times 10^{5}$	$8.09\times 10^{3}$	$1.83\times 10^{3}$
	Rank	2	1	6	7	5	3	4
F59	Ave	1600.71	1604.53	$1.78\times 10^{3}$	$1.86\times 10^{3}$	$1.75\times 10^{3}$	$1.81\times 10^{3}$	$1.69\times 10^{3}$
	std	$2.69\times 10^{-1}$	$2.15\times 10^{1}$	$1.03\times 10^{2}$	$1.04\times 10^{2}$	$1.10\times 10^{2}$	$1.87\times 10^{2}$	$8.49\times 10^{2}$
	Rank	1	2	5	7	4	6	3
F60	Ave	2100	2100	$5.88\times 10^{3}$	$5.52\times 10^{4}$	$1.03\times 10^{4}$	$3.00\times 10^{3}$	$2.34\times 10^{3}$
	std	0.338	3.06	$4.03\times 10^{3}$	$5.44\times 10^{4}$	$4.62\times 10^{3}$	$7.36\times 10^{2}$	$2.35\times 10^{2}$
	Rank	1	2	5	7	6	4	3
F61	Ave	2266.42	$2.27\times 10^{3}$	$2.42\times 10^{3}$	$2.32\times 10^{3}$	$2.36\times 10^{3}$	$2.30\times 10^{3}$	$2.31\times 10^{3}$
	std	$4.59\times 10^{1}$	$4.48\times 10^{1}$	$2.45\times 10^{2}$	$2.17\times 10^{1}$	$1.61\times 10^{2}$	$1.60\times 10^{1}$	$2.05\times 10^{1}$
	Rank	1	2	7	5	6	3	4
F62	Ave	2574.29	$2.59\times 10^{3}$	$2.69\times 10^{3}$	$2.76\times 10^{3}$	$2.74\times 10^{3}$	$2.74\times 10^{3}$	$2.74\times 10^{3}$
	std	$1.09\times 10^{2}$	$1.14\times 10^{2}$	$1.32\times 10^{1}$	$6.95\times 10^{1}$	$1.14\times 10^{1}$	$6.94\times 10^{1}$	$2.84\times 10^{1}$
	Rank	1	2	3	7	4	6	5
F63	Ave	2908	$2.92\times 10^{3}$	$2.93\times 10^{3}$	$2.95\times 10^{3}$	$2.94\times 10^{3}$	$2.92\times 10^{3}$	$2.93\times 10^{3}$
	std	19.44	$2.24\times 10^{1}$	$2.85\times 10^{1}$	$6.73\times 10^{1}$	$1.70\times 10^{1}$	$2.30\times 10^{1}$	$2.56\times 10^{1}$
	Rank	1	2	5	7	6	3	4
	Ave Rank	1.5	1.7	4.5	6.1	5.0	3.6	3.5

**Table 6:** Model parameters.

Parameter Symbol	Value
Population size *N*	30
Number of iterations *T*	1000
Weighting of costs α	10,100,10,50
Starting point coordinates	(200,100,150), (50,50,100)
Target point coordinates	(800,800,150), (350,360,100)

**Table 7:** Model initialization and average cost.

Scenario	((X,Y,Z),R)	ISCHNOA	NOA	SCHO	WOA	GWO	HBA	EO
1	((400,500,200),50),((500,350,200),50)							
	((600,200,200),40)	9310	9556	9265	9321	9258	10,800	9256
2	((325,350,200),50),((400,550,200),50)							
	((500,550,200),40),((600,550,200),40)	10,090	10,390	10,610	10,433	10,435	11,090	10,460
3	((325,350,200),50),((400,550,200),50)							
	((500,325,200),40),((550,650,200),40)							
	((625,500,200),40)	9362	11,113	10,580	11,320	10,440	9822	10,430
4	((350,200,200),40),((400,500,200),50)							
	((500,350,200),50),((600,200,100),40)							
	((650,750,150),50),((700,550,200),40)	10,460	10,840	11,140	11,750	11,010	11,710	10,980
5	((150,250,100),15),((250,125,200),15)	4420	4410	4406	4379	4397	4383	4379
6	((125,200,100),20),((175,275,100),20)							
	((200,125,200),15),((250,200,100),15)	5155	6273	5997	5416	5465	5837	5391
7	((100,200,100),20),((150,350,100),20)							
	((175,250,100),15),((200,125,100),15)							
	((275,200,100),15),((300,300,100),20)	6288	7240	7758	6482	6397	8221	6478
8	((100,200,100),20),((150,350,100),20)							
	((175,250,100),15),((200,125,100),15)							
	((250,200,100),20),((250,300,100),15)	7507	9327	8353	8506	9178	10224	8032

**Table 8:** Sensitivity analysis of the cost of taking the value of *K* in ISCHNOA for eight scenarios.

Scenario	Cost (*k* = 150)	Cost (*k* = 200)	Cost (*k* = 250)	Cost (*k* = 300)	Cost (*k* = 350)
1	9341	9295	9310	9301	9321
2	10,069	10,041	10,090	10,295	10,263
3	9572	9397	9362	9410	9378
4	10,359	10,265	10,460	10,540	10,332
5	4410	4400	4420	4416	4413
6	5186	5162	5155	5175	5130
7	6528	6597	6288	6562	6683
8	7524	7418	7507	7561	7461

## Data Availability

Available under request.
